# A Novel Chitin‐Based Purification System Using GAL1 Fusion Tags: Enhancing Recombinant Protein Production While Retaining Biological Activity

**DOI:** 10.1111/1751-7915.70157

**Published:** 2025-05-15

**Authors:** Yao‐Kuang Tseng, Yun‐Heng Lu, Yun Liu, Zhi‐Wei Weng, Yu‐Tzu Lin, Chih‐Hsuan Tsai, Yueh‐Lung Wu, Rong‐Nan Huang

**Affiliations:** ^1^ Department of Entomology National Taiwan University Taipei Taiwan; ^2^ Department of Microbiology and Immunology National Cheng Kung University Tainan Taiwan; ^3^ Master Program for Plant Medicine National Taiwan University Taipei Taiwan

**Keywords:** affinity chromatography, chitin, Galectin‐1, Melittin, recombinant protein purification

## Abstract

Efficient and economical purification methods are crucial for the commercial production of recombinant proteins with biomedical applications. In this study, we developed an affinity chromatography system that leverages the polysaccharide‐binding properties of galectin‐1 (GAL1) as a protein tag. The known GAL1‐binding material, chitin, was used as the purification matrix. Melittin (MELT), a bee venom peptide known for its antimicrobial and anti‐inflammatory properties with commercial potential, was chosen to validate this system. The GAL1–MELT fusion protein was expressed in *Escherichia coli* ( *E. coli* ) and successfully purified using a chitin‐based matrix with sodium dodecyl sulfate (SDS) as a removable eluant. This method demonstrated higher purification efficiency compared to the His‐tag/Ni‐NTA approach, indicating that the GAL1/chitin system could serve as a superior alternative. The GAL1–MELT fusion protein retained strong antibacterial and anti‐inflammatory activities, as well as collagen content modulation effects, confirming that MELT maintained its bioactivity. Apart from that, the GAL1–DsRed fusion protein was used as an additional protein target to evaluate the efficiency of the chitin‐based column. Notably, all experiments were conducted without tag cleavage, showing that enzyme treatments for MELT isolation were unnecessary. This study highlights the potential of GAL1–polysaccharide interactions as a cost‐effective and highly efficient alternative method for recombinant protein purification.

## Graphical abstract

A chitin‐based affinity chromatography system using GAL1 as a fusion tag efficiently purifies recombinant proteins while retaining their biological activity, offering a cost‐effective alternative to conventional purification methods.

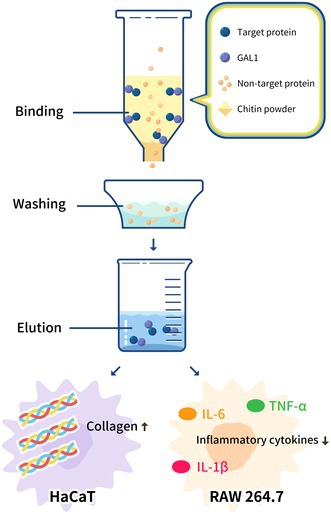

## 1 Introduction

Advancements in genetic engineering have significantly promoted the research on recombinant proteins, especially the development of efficient purification methods. However, the isolation of target proteins with high specificity from complex mixtures remains challenging. Affinity chromatography is widely used for protein purification because of its specificity and efficiency in utilising matrices that selectively bind to tagged proteins (Rodriguez et al. 2020). Common fusion tags, such as His‐tag, GST‐tag, and MBP‐tag, have facilitated recombinant protein purification but have limitations in terms of cost, solubility enhancement, and tag removal (Lichty et al. 2005). This has led to a growing demand for novel fusion tags and matrices that achieve a balance of efficiency, scalability, and cost. A previous study presented the Spy&Go system (Khairil Anuar et al. 2019), describing the development of a novel fusion tag and purification system.

Here, we propose galectin‐1 (GAL1), a β‐galactoside‐binding protein derived from mammals, that specifically recognises carbohydrate structures, particularly N‐acetyllactosamine (Leppänen et al. 2005; Stowell et al. 2004, 2008). Additionally, GAL1 contains a carbohydrate‐recognition domain (CRD) that interacts with N‐acetylglucosamine (Camby et al. 2006; Pasmatzi et al. 2019), a structural unit similar to chitin. This unique binding property has expanded the potential applications of GAL1 in protein purification. Previous studies have demonstrated that GAL1 can bind to chitosan membranes, enhancing cell adhesion and promoting cell growth, indicating its potential for biomedical and tissue engineering applications (Chang et al. 2004). Furthermore, feeding GAL1‐containing leaves to diamondback moths (*Plutella xylostella*) disrupted their peritrophic membranes, indicating specific interactions between GAL1 and chitin structures (Chen et al. 2009). These findings suggest that GAL1 can serve as a promising fusion tag to improve the solubility and stability of recombinant proteins and facilitate their purification (Pasek et al. 2010). In this study, we propose a purification system using chitin, an abundant and cost‐effective material, as a matrix to evaluate the performance of GAL1 as a fusion tag for protein purification.

Melittin, a bee venom peptide consisting of 26 amino acids with an α‐helical structure, was chosen as the test protein to validate this system (Terwilliger et al. 1982). Melittin is a major active component of bee venom, comprising 40%–60% of its dry protein content (Chen et al. 2016). The amphipathic nature of melittin allows it to be inserted into lipid bilayers, creating pores that increase membrane permeability and induce cell lysis (Guha et al. 2021). Melittin exhibits various biological activities, including antibacterial, anti‐inflammatory, antifungal, and antitumor effects, and has been widely studied in the biomedical, food, and cosmetic fields (Rady et al. 2017; Choi et al. 2014; Jo et al. 2012; Liu et al. 2008; Shin et al. 2013; Son et al. 2007; Yang et al. 2014; Wehbe et al. 2019; Isidorov et al. 2023; El‐Wahed et al. 2021; Khalifa et al. 2021). However, natural melittin extraction is labor‐intensive, yields low quantities, and requires venom secretion from a large number of worker bees. This renders large‐scale production challenging. Therefore, we utilised *Escherichia coli* to express the GAL1–MELT fusion protein, which was purified using a chitin‐based matrix to assess the potential of GAL1 as a fusion tag for recombinant protein purification.

The purified GAL1–MELT fusion protein was subjected to multiple functional assays, including antibacterial activity (growth inhibition of *E. coli* and *Bacillus subtilis* ), anti‐inflammatory gene expression analysis (qPCR of *IL‐1β*, *IL‐6*, and *TNF‐α* in RAW 264.7 macrophages), and collagen production assessment (collagen content in HaCaT cells). The results demonstrated that GAL1 enhanced the purification efficiency compared to His‐tag purification methods, and the purified melittin retained its biological activity. Furthermore, to demonstrate the applicability of this purification strategy to different types of target proteins, we utilised the GAL1–DsRed fusion protein and confirmed that the chitin‐based system can be applied to a variety of proteins. This study highlights the potential of GAL1–polysaccharide interactions for the development of an efficient and cost‐effective purification system for recombinant protein production.

## 2 Experimental Procedures

### 2.1 Bacterial Culturing and Protein Overexpression

*Escherichia coli* strains DH5α and M15 (Yeastern Biotech, New Taipei City, Taiwan) were used for plasmid amplification and protein overexpression. Bacterial cultures were grown in Luria‐Bertani (LB) broth (Bioman, New Taipei City, Taiwan) following the manufacturer's instructions. LB agar plates were prepared with 1.5% agar (Alpha Biosciences, Maryland, USA) and supplemented with ampicillin (100 μg/mL) for selection. Cultures were incubated at 37°C with shaking at 200 rpm for 16 h. Protein overexpression was induced by adding 1 mM isopropyl β‐D‐1‐thiogalactopyranoside (IPTG) when bacterial cultures were re‐cultured to OD_600_ = 0.5 and shaking for 4 h at 37°C. Bacterial cultures were centrifuged at 3000× *g* for 15 min, and pellets were washed twice with deionised water and stored at −20°C for subsequent use.

### 2.2 Plasmid Construction and Transformation

The GAL1 gene was amplified from Chinese hamster ovary cell DNA using the T&A Cloning Vector Kit (Yeastern Biotech, New Taipei City, Taiwan) and inserted into the pBacPAK8 vector (Takara, Kusatsu, Japan) at the *XhoI* and *XbaI* sites. The MELT gene was reverse‐transcribed from honeybee RNA into cDNA and inserted into pBacPAK8 at the *XhoI* and *SmaI* sites. The DsRed sequence was amplified from the pABpR vector. PCR primers were designed with restriction sites and listed in Table 1. Among them, the linker sequence (LVPRGS) was designed into the MELT primer and DsRed primer, as a preparation for future studies of enzyme cleavage with thrombin and factor Xa (Jenny et al. 2003). The PCR amplification followed a program of 95°C for 5 min (pre‐denaturation), 30 cycles of 95°C for 30 s, 55°C for 30 s, and 72°C for 1 min, followed by a final extension at 72°C for 5 min. PCR products were purified using the GeneHlow Gel/PCR Kit (Geneaid, New Taipei City, Taiwan), and plasmid DNA was extracted using the Presto Mini Plasmid Kit (Geneaid, New Taipei City, Taiwan). The GAL1–Linker–MELT fusion sequence was ligated into the pQE_30 vector using *BamHI* and *PstI* sites, and the GAL1–Linker–DsRed fusion sequence was ligated into the pQE_30 vector using *BamHI* and *HindIII* sites. Ligation was performed overnight at 4°C using the Takara DNA Ligation Kit. The construct was transformed into DH5α cells via heat shock at 42°C for 45 s and plated on LB agar containing ampicillin (100 μg/mL) for overnight incubation at 37°C.

### 2.3 Protein Extraction and Immunoblotting

Bacterial pellets were resuspended in binding buffer (1X PBS, pH 7.4) containing protease inhibitors and sonicated for 5 min (30% amplitude, 2‐s pulses). Lysates were centrifuged at 3000× *g* for 20 min at 4°C, and the supernatant was collected. GAL1–MELT protein and GAL1–DsRed protein samples were mixed with 2X Laemmli buffer and heated at 98°C for 15 min. Proteins were separated by SDS‐PAGE (15% gel) and transferred to PVDF membranes. Membranes were blocked with 5% non‐fat milk in TBST (1X) for 1 h, incubated overnight at 4°C with a primary anti‐6X HIS antibody (1:5000 dilution) and washed with TBST (1X). Secondary antibody (1:10,000 dilution) was applied for 1 h at room temperature, and proteins were visualised using chemiluminescence. The relative protein level was calculated based on the graphical analysis from ImageJ.

### 2.4 Chitin Pull‐Down Assay and Chitin‐Based Column Purification

*Escherichia coli* M15 strain was cultured in 3 mL of LB broth at 37°C overnight for protein overexpression. After sonication, 2 mg of crude protein extracts were incubated with chitin powder (Tokyo Chemical Industry) at 4°C for 1 h or 24 h, with gentle shaking throughout the incubation to facilitate binding. 5 mg of chitin powder was added into a 1.5 mL tube for binding. After incubation, the chitin pellet was washed three times with binding buffer (1X PBS, pH 7.4) and centrifuged at 500 rpm for 5 min at 4°C to remove unbound or excess proteins. For protein elution, PBS (1X) containing 0.1%–2% SDS was added to fully resuspend the chitin pellet. The pellet was then mixed with 100 μL of sample buffer, followed by heat treatment at 98°C for 15 min. The supernatant was collected and subjected to SDS‐PAGE and western blot analysis. For the chitin column‐based pull‐down assay, 55 mg of chitin powder (27.5 mg chitin/mg crude protein) was added into a 16 cm‐height syringe‐like column and mixed with the protein extracts. The mixture was incubated at 4°C with gentle shaking, and the column was sealed with parafilm to prevent leakage. After incubation, the cap was opened to allow the liquid to flow out naturally, and unbound proteins were removed by washing. Washing was performed by adding three times the volume of chitin of 1X PBS for three times. Elution was performed by the flow‐through with elution buffer (binding buffer containing 0.1% SDS). The buffer in each elution was allowed to remain in the column for 5 min. After elution, all purified protein samples were immediately treated with the SDS‐Out SDS Precipitation Kit (Thermo Fisher, Waltham, USA) to remove residual SDS before downstream analysis. The protein concentration was determined using the Bio‐Rad Protein Assay (Bio‐Rad, California, USA) based on the Bradford method. Briefly, 10 μL of each protein sample was mixed with 200 μL of Bio‐Rad Protein Assay Dye Reagent in a 96‐well plate. After incubation at room temperature for 5 min, absorbance was measured at 595 nm using a microplate reader. A series of BSA standards (50–500 μg/mL) was used to build a standard curve to quantify the protein concentration. All measurements were performed in triplicate for accuracy. The protein purified yield and purity were quantified with ImageJ. Briefly, the original SDS‐PAGE gel image was used to analyse total protein signal (pixel density) of each lane (S_total_). The signal of the band indicating fusion protein was also analysed (S_fusion_). The purity was calculated as the following equation: purity = S_fusion_/S_total_ × 100%.

### 2.5 Cell Culture and RNA Extraction for RT‐qPCR

RAW 264.7 and HaCaT cells were cultured in high‐glucose DMEM with 10% FBS at 37°C in a 5% CO_2_ incubator. The concentration of the MELT was standardised based on its molar concentration (M) in accordance with the GAL1–MELT fusion protein content. Each reagent was added into cultured cells (3 × 10^5^ cells/per 24‐well) after removing the medium and washing with PBS (1X). At the end of experimental times, the cells were treated with TrypLE (Thermo Fisher, Waltham, USA) for 5 min at 37°C, and the resulting suspended cells were collected for extracting RNA. Total RNA was extracted using the Total RNA Mini Kit (Geneaid, New Taipei City, Taiwan) and quantified using a nano‐drop spectrophotometer. RNA (500 ng) was reverse‐transcribed using the PrimeScript RT Reagent (Takara, Kusatsu, Japan). RT‐qPCR was done in a 20 μL reaction with 5 μL cDNA, 10 μL SensiFAST SYBR Hi‐ROX mix (Meridian, Korea), and 0.8 μL primers at 0.5 μM final concentration with a qTOWER3G machine under the following conditions: 95°C for 5 s, 60°C for 10 s, and 72°C for 20 s, for 40 cycles.

### 2.6 Anti‐Bacterial Assay

Gram‐negative *E. coli* DH5α and gram‐positive *Bacillus subtilis* (Bs) strains were used for antibacterial testing. Bacteria were cultured overnight at 37°C in LB broth, diluted to OD_600_ = 0.1, and adjusted to 2 × 10^5^ CFU/mL in fresh LB broth. A 100 μL of bacterial suspension was added to each well of a 96‐well plate. The GAL1–MELT fusion protein was subjected to a two‐fold serial dilution from 100 μg/mL to 1.56 μg/mL and determined the minimum inhibitory concentration (MIC) comparing with commercial MELT (Sigma, Missouri, USA; cMELT). GAL1 and PBS (1X) were set as negative controls, and LB broth alone was set as a control. Plates were incubated at 35°C for 16 h, and OD_600_ was measured using a microplate reader to represent bacterial growth. All experiments included three replicates.

### 2.7 Anti‐Inflammatory Assay

RAW 264.7 cells were seeded at 3 × 10^5^ cells per well in 24‐well plates and cultured in high‐glucose DMEM with 10% FBS at 37°C in 5% CO_2_ overnight until 70%–80% confluence. Cells were treated with 1 μg/mL lipopolysaccharide (LPS) and either 0.33 μM cMELT, 0.33 μM GAL1–MELT, or 0.7 μM GAL1. DMEM was used as a control. After 4 h, cells were collected and analysed for *TNF‐α*, *IL‐1β*, and *IL‐6* mRNA expression levels via RT‐qPCR. The primer sequences are listed in Table 1. mRNA levels were calculated using the ΔΔ*C*_*t*_ method relative to the control (Livak and Schmittgen 2001).

### 2.8 Collagen Assay and Cell Sensitization Test

HaCaT cells were seeded at 3 × 10^5^ cells per well in 24‐well plates and cultured in high‐glucose DMEM with 10% FBS at 37°C in 5% CO_2_ overnight. The cells were treated with cMELT (0.13 μM, 0.26 μM), GAL1–MELT (0.13 μM, 0.26 μM), or GAL1 (0.28 μM, 0.56 μM), and DMEM was used as a negative control. After 24 h, the supernatants were collected, and collagen levels were measured using a collagen assay kit (Sigma‐Aldrich, Missouri, USA) according to the manufacturer's instructions. Cells RNA was extracted and analysed for *Fos* and *FosL1* mRNA expression levels via RT‐qPCR. The primer sequences are listed in Table 1. The expression levels of *Fos* and *FosL1* mRNA were normalised to GAPDH and analysed using the ΔΔ*C*_*t*_ method.

### 2.9 Statistical Analysis

Statistical analyses were performed using GraphPad Prism 9.0. Significant differences (*p* < 0.05) were determined using Student's *t*‐test for comparisons between two groups or one‐way ANOVA for multiple group comparisons. Results are presented as mean ± SD, and all experiments were performed in triplicate (*n* = 3). Significant differences among groups are indicated with different letters in the figures to denote statistical significance.

## 3 Results

### 3.1 Expression and Purification of GAL1–MELT Fusion Protein With Specific Binding and Elution Efficiency

**FIGURE 1:**
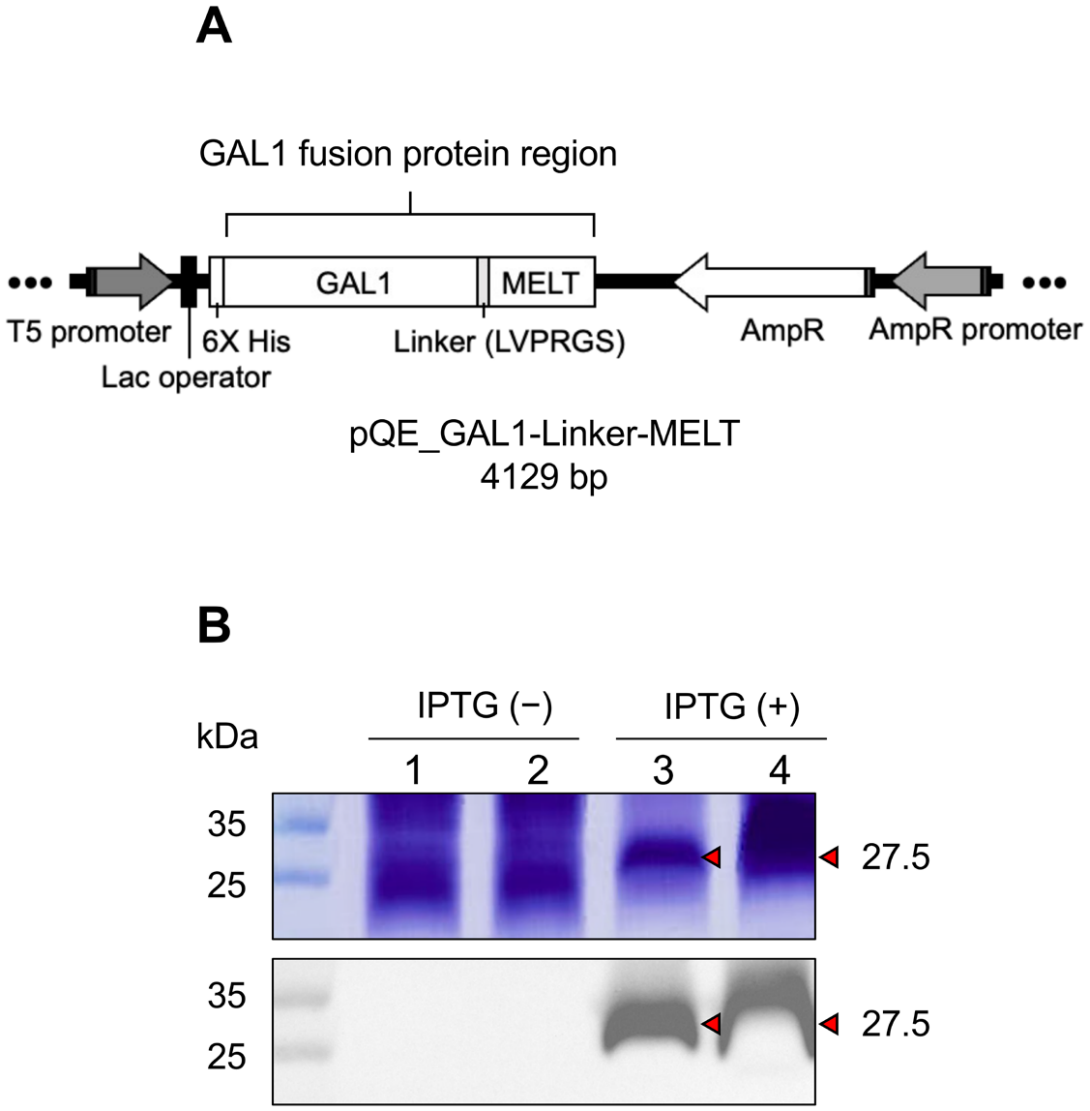
Construction and expression of the GAL1–MELT fusion protein. (A) Diagram showing the genetic structure of the pQE_GAL1–Linker–MELT construct (4129 bp). (B) Expression of the GAL1–MELT fusion protein (27.5 kDa) in *E. coli* M15 after 4 h of IPTG induction. Lanes 1 and 2 show uninduced samples, while lanes 3 and 4 show IPTG‐induced samples. Protein samples were analysed by SDS‐PAGE and visualised with Coomassie staining and Western blotting.

**FIGURE 2:**
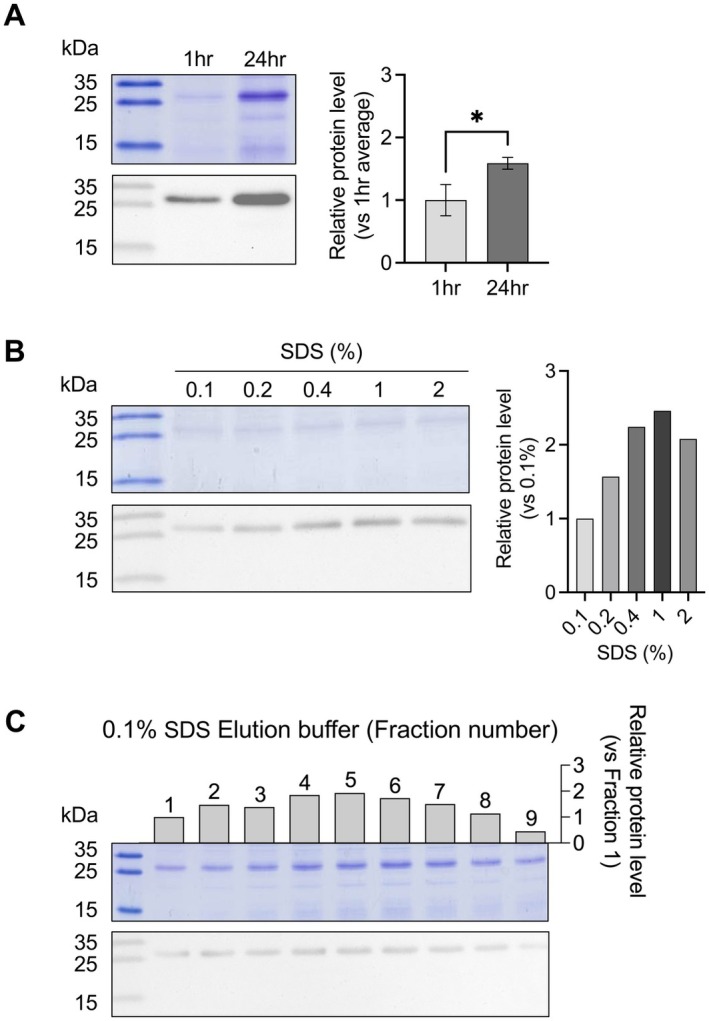
Affinity of GAL1–MELT fusion protein for chitin and elution with SDS solution. (A) Pull‐down assay showing GAL1–MELT crude extracts incubated with chitin powder for 1 or 24 h at 4°C. The GAL1–MELT fusion protein (27.5 kDa) was detected by SDS‐PAGE and Western blotting. Statistical analysis was done using Student's *t*‐test, with error bars indicating standard deviation (SD). Asterisks indicate significant differences (*p* < 0.05). *p < 0.05. (B) Elution of GAL1–MELT fusion protein with SDS solutions at concentrations of 0.1%, 0.2%, 0.4%, 1%, and 2%. (C) Elution using 0.1% SDS, with nine 1 mL fractions collected and analysed by SDS‐PAGE and western blotting. Protein levels in each fraction are shown as bar charts.

In this study, we constructed a pQE_GAL1–Linker–MELT expression vector (Figure 1A) and expressed it in *E. coli* M15 cells. SDS‐PAGE analysis showed a protein band at approximately 27.5 kDa after IPTG induction, indicating the successful expression of the GAL1–MELT fusion protein (Figure 1B). Western blotting confirmed that the band corresponded to the target fusion protein. To examine the binding specificity, we conducted a chitin pull‐down assay, which revealed a clear protein band at 27.5 kDa (Figure 2A), consistent with specific binding to chitin. Anti‐His tag western blotting further verified this result by showing a single band. The binding was also observed to be time‐dependent. The elution efficiency was tested using different concentrations of SDS. The results indicated that 0.1% SDS was sufficient for the effective elution of the fusion protein, with stronger signals at higher SDS concentrations (Figure 2B). Subsequent column purification confirmed the presence of the protein in multiple fractions, with the strongest signal detected in fractions 3–6, and western blotting confirmed the specificity of the eluted proteins (Figure 2C). The relative protein level was quantified by analysing the pixel density of the target protein band on the SDS‐PAGE gel image using ImageJ. In summary, the GAL1–MELT fusion protein was successfully expressed, and it demonstrated consistent binding and elution properties, supporting its potential use in recombinant protein purification.

### 3.2 Comparison of GAL1–MELT Fusion Protein Purification and Evaluation of Its Antibacterial Activity

**FIGURE 3:**
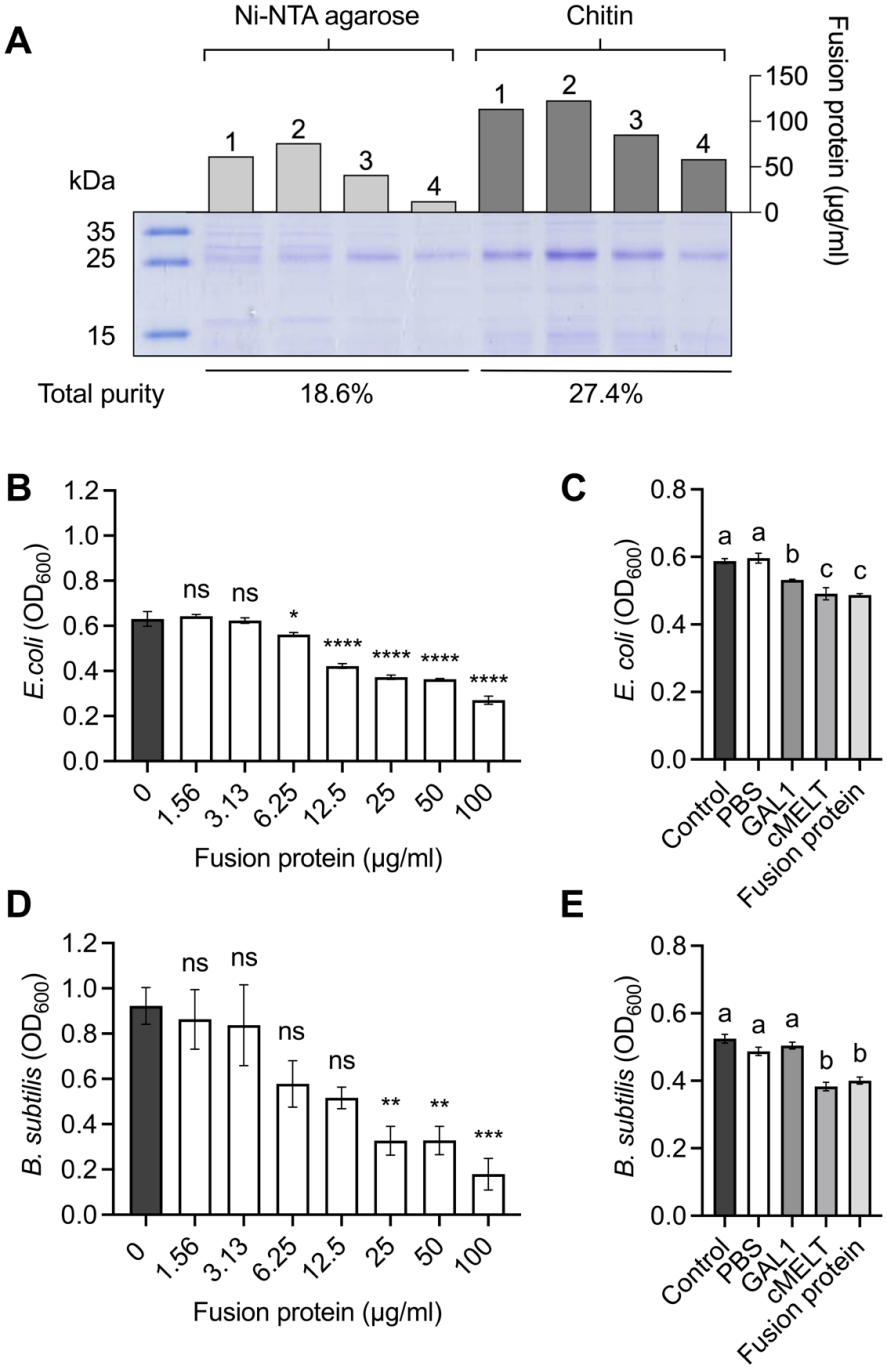
Purification efficiency and antibacterial activity of GAL1–MELT fusion protein. (A) Comparison of GAL1–MELT purification using Ni‐NTA and chitin columns. A total of 500 μg of protein and 1 g of resin were used for purification. Four fractions (1, 2, 3, and 4) were collected, and protein concentrations were measured using a NanoDrop spectrophotometer and presented as bar charts. (B, D) Minimum inhibitory concentration (MIC) of GAL1–MELT fusion protein for *E. coli* DH5α (6.25 μg/mL) and *Bacillus subtilis* (25 μg/mL). Statistical significance was determined by Student's *t*‐test. *p < 0.05; ^**^ *p* < 0.01; ^***^ *p* < 0.001; ^****^ *p* < 0.0001. (C, E) Antibacterial activity of GAL1–MELT fusion protein compared to PBS (1X) (negative control), GAL1 (at MIC‐equivalent concentrations), and cMELT (positive control). Data are shown as mean ± SD. One‐way ANOVA was used to test significance (*p* < 0.05), with different letters indicating significant differences among groups.

The pQE_GAL1–Linker–MELT expression vector was expressed in *E. coli* M15 cells, and 500 μg of crude protein extract was subjected to purification using both chitin affinity columns and Ni‐NTA columns. SDS‐PAGE analysis of fractions 1 to 4 from both purification methods revealed distinct protein bands at 27.5 kDa (Figure 3A), indicating successful purification of the GAL1–MELT fusion protein using both approaches. The Ni‐NTA columns used 1 mL of 50 mM imidazole, and the chitin‐based column used 0.1% SDS elution buffer. Protein purity was assessed using the ratio of the pixel density of the total staining area to that of the target protein staining area on the SDS‐PAGE gel as determined by ImageJ. The protein purities obtained using the Ni‐NTA and chitin affinity columns were 18.6% and 27.4%, respectively. The purified protein yield eluted with 1–4 fractions was 35.5 μg using the Ni‐NTA columns and 104.5 μg using the chitin affinity columns (calculation method: purified protein yield = protein purity × total amount of elution protein). To evaluate the antibacterial activity of the GAL1–MELT fusion protein, Thermo SDS‐Out was used to remove residual SDS from the protein samples to avoid interference in subsequent assays. The GAL1–MELT fusion protein was incubated with *E. coli* and *B. subtilis* cultures at 35°C for 16 h. The results showed that significant inhibition of *E. coli* growth was observed at GAL1–MELT concentrations of 6.25 μg/mL and above, while for *B. subtilis* , significant inhibition required 25 μg/mL, corresponding to the minimum inhibitory concentration (MIC) for each strain (Figure 3B,D).

Further comparisons of antibacterial activity were conducted using PBS (1X), GAL1, cMELT, and the GAL1–MELT fusion protein. The results showed that both GAL1–MELT and cMELT exhibited significant antibacterial effects against *E. coli* and *B. subtilis* (Figure 3C,E). Although GAL1 also showed antibacterial activity against *E. coli* , its effect was less pronounced than that of GAL1–MELT and cMELT. In contrast, GAL1 and PBS (1X) showed no significant inhibitory effect on *B. subtilis* . In summary, the GAL1–MELT fusion protein was effectively purified using chitin affinity and Ni‐NTA columns. It demonstrated antibacterial activity identical to cMELT, suggesting that the GAL1 tag did not influence its protein activity.

### 3.3 Suppression of Pro‐Inflammatory Cytokine Expression by GAL1–MELT Fusion Protein

**FIGURE 4:**
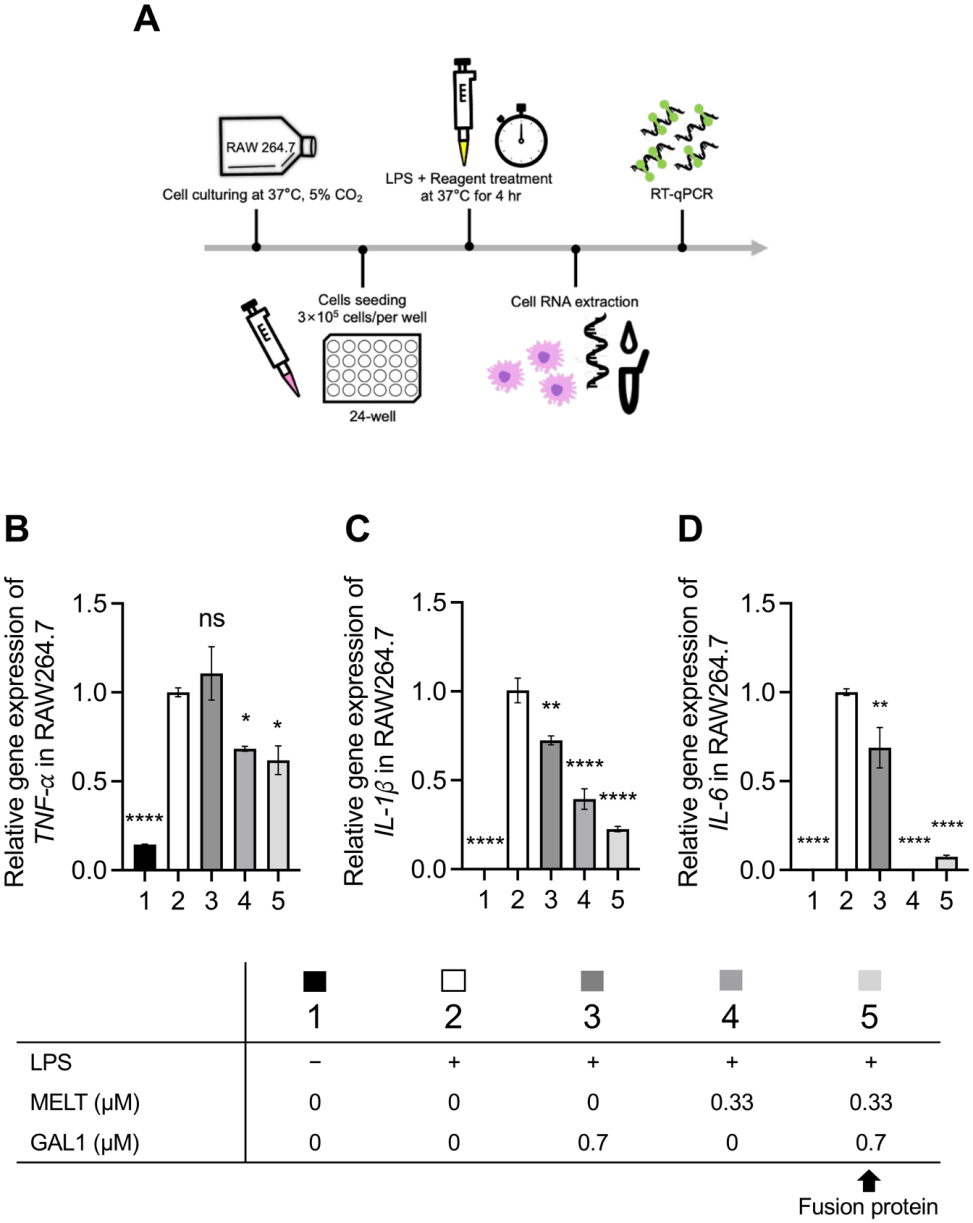
Anti‐inflammatory activity of GAL1–MELT fusion protein. (A) Diagram of RT‐qPCR analysis for evaluating the anti‐inflammatory response in RAW 264.7 cells treated with purified bee venom protein. RAW 264.7 cells were treated with LPS (1 μg/mL) for 4 h. The mRNA levels of *TNF‐α* (B), *IL‐1β* (C), and *IL‐6* (D) were measured by qPCR. Sample concentrations are shown in the lower panel. Statistical analysis was performed using Student's *t*‐test (*p* < 0.05). Error bars indicate SD. *p < 0.05; ^**^ *p* < 0.01; ^****^ *p* < 0.0001.

The anti‐inflammatory activity of the GAL1–MELT fusion protein was assessed by measuring its ability to inhibit pro‐inflammatory cytokines TNF‐α, IL‐1β, and IL‐6 in RAW 264.7 macrophages stimulated with LPS (1 μg/mL). A schematic diagram of the experimental procedure is shown in Figure 4A. Compared to the blank and DMEM control groups (0 μM), treatment with GAL1–MELT and cMELT for 4 h reduced TNF‐α‐ mRNA levels, with GAL1–MELT showing stronger suppression than GAL1 alone (Figure 4B). Both GAL1–MELT and cMELT also suppressed *IL‐1* and *IL‐6* expression, while GAL1 alone showed moderate inhibition (Figure 4C,D). This effect may be related to the immunomodulatory properties of GAL1 (Mansour et al. 2022). The fusion protein showed stronger suppression of inflammatory cytokine expression compared to GAL1 alone. These findings indicate that the GAL1–MELT fusion protein has anti‐inflammatory activity, supporting its potential for applications in inflammatory regulation and treatment.

### 3.4 Effects of GAL1–MELT Fusion Protein on Collagen Expression and Sensitisation Potential in HaCaT Cells

**FIGURE 5:**
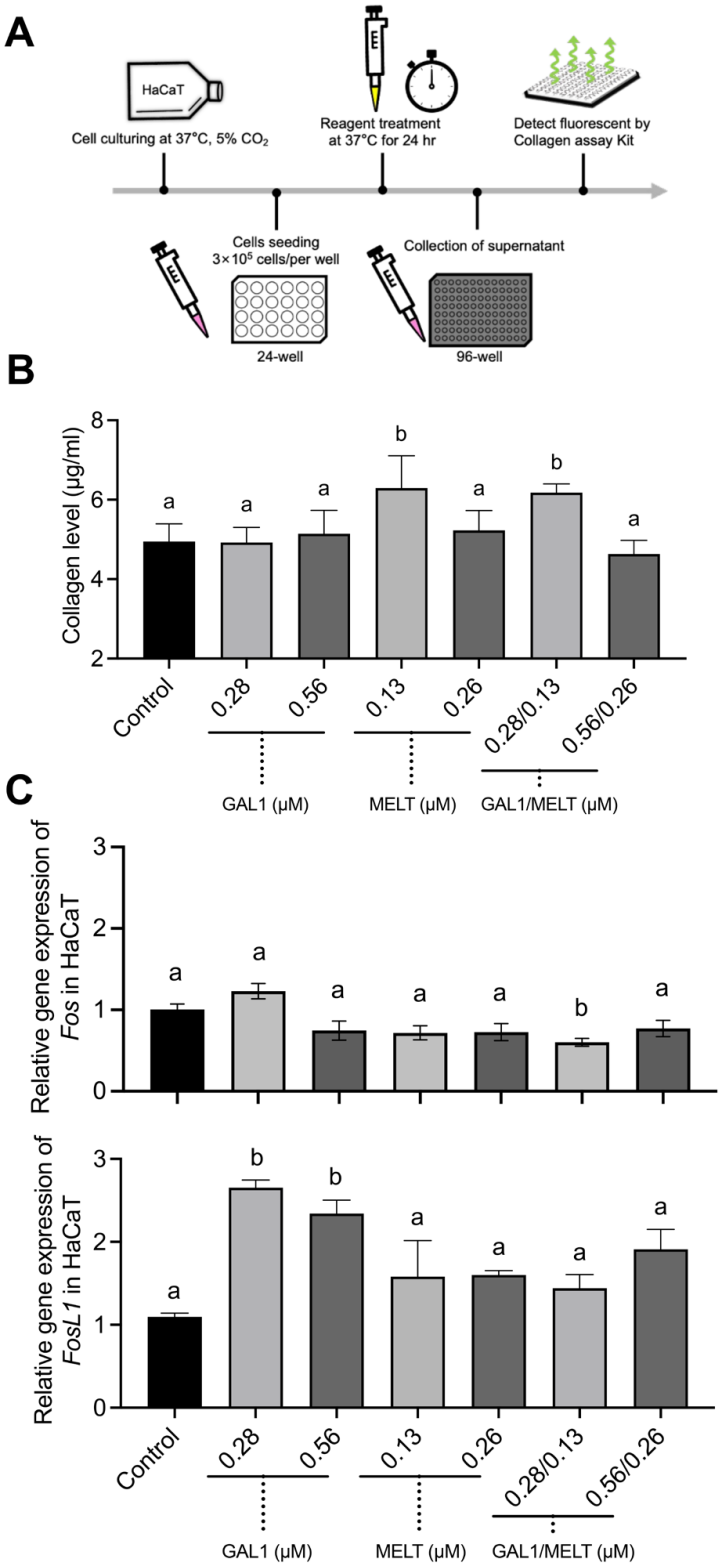
Collagen assay and gene expression in HaCaT Cells. (A) This illustration depicts the experimental workflow for collagen quantification and qPCR analysis of cellular sensitisation responses in HaCaT cells. (B) Collagen levels in the supernatant after 24 h of treatment were measured using a Collagen Assay Kit, with fluorescence detection at λex = 375 nm and λem = 465 nm. Collagen concentrations were calculated using a standard curve. (C) *Fos* and *FosL1* mRNA expression levels were measured by qPCR. Data are presented as mean ± SD. One‐way ANOVA was used to test significance. Significant differences were observed in the GAL1 group (*p* < 0.01), while no significant differences were found in other groups (*p* > 0.05). Different letters indicated the existance of significant differences.

The effects of the GAL1–MELT fusion protein on type I collagen expression and sensitization‐related gene expression (*Fos* and *FosL1*) were assessed in HaCaT cells after 24 h of co‐culture. A schematic diagram of the experimental procedure is shown in Figure 5A. The results showed that at 0.26 μM concentration, both the GAL1–MELT fusion protein and cMELT had no significant effect on collagen expression compared to the control group. Similarly, 0.28 μM and 0.56 μM GAL1 did not significantly alter collagen levels. However, at 0.13 μM, both the GAL1–MELT fusion protein and cMELT increased type I collagen expression (Figure 5B). To evaluate the sensitization potential, *Fos* and *FosL1* expression levels were measured. The results indicated that at 0.26 μM and 0.13 μM, neither the GAL1–MELT fusion protein nor cMELT significantly increased *Fos* or *FosL1* expression compared to the negative control. However, GAL1 at 0.56 μM and 0.28 μM significantly increased *FosL1* expression but did not elevate *Fos* expression (Figure 5C). In conclusion, the GAL1–MELT fusion protein promoted type I collagen expression at low concentrations without inducing sensitisation. These findings suggest a potential application for skin repair without triggering hypersensitivity reactions.

### 3.5 Validation of the GAL1–Chitin Purification System Using an Additional Target Protein

**FIGURE 6:**
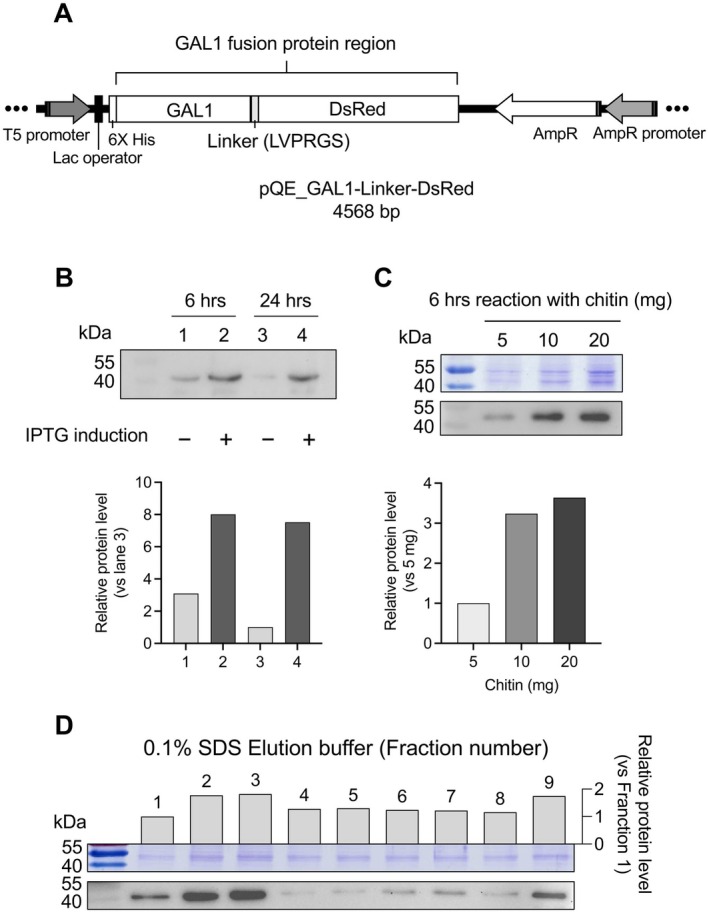
GAL1–DsRed fusion protein purified with chitin‐based column successfully. (A) Diagram showing the genetic structure of the pQE_GAL1–DsRed construct (4568 bp). (B) Expression of the GAL1–DsRed fusion protein (42.8 kDa) in *E. coli* M15 after 6 h and 24 h of IPTG induction. Lanes 1 and 3 show uninduced samples, while lanes 2 and 4 show IPTG‐induced samples. Protein samples were analysed by SDS‐PAGE and visualised with Coomassie staining and western blotting. (C) Pull‐down assay showing GAL1–DsRed crude extracts incubated with chitin powder (5, 10, 20 mg) for 6 h at RT. The GAL1–DsRed fusion protein (42.8 kDa) was detected by SDS‐PAGE and western blotting. (D) Elution using 0.1% SDS, with nine 1 mL fractions collected and analysed by SDS‐PAGE and western blotting. Protein levels in each fraction are shown as bar charts.

To evaluate the reproducibility and versatility of the GAL1–chitin purification system, we selected DsRed, a fluorescent reporter protein, as a second fusion target. The pQE_GAL1–Linker–DsRed expression vector was constructed (Figure 6A) and expressed in *E. coli* M15 cells. Crude protein extracts (5 mg) were subjected to chitin‐based column purification. SDS‐PAGE and western blot analyses revealed a clear protein band at 42.8 kDa following IPTG induction, confirming the successful expression of the GAL1–DsRed fusion protein (Figure 6B). The relative protein level was quantified by analysing the pixel density of the target protein band on the western blot signalling image using ImageJ. To assess the binding efficiency and specificity, a chitin pull‐down assay was performed using different chitin amounts (5, 10, and 20 mg). A strong, dose‐dependent signal at 42.8 kDa was observed (Figure 6C), indicating effective binding of GAL1–DsRed to chitin. Furthermore, elution with 0.1% SDS yielded the majority of purified protein in fractions 1–9, as visualised by SDS‐PAGE gel and quantified using ImageJ (Figure 6D). These results confirmed that the GAL1–chitin system is applicable to other recombinant proteins beyond melittin and supports its broader utility in protein purification workflows.

## 4 Discussion

This study showed that the GAL1 tag system significantly improved purification efficiency compared to the commonly used HIS tag (Figure 3A), while preserving the biological activity of melittin (Figures 3, 4, 5). The larger fusion protein GAL1–DsRed was also successfully purified using the GAL1 tag and chitin‐based purification system (Figure 6). Our results effectively addressed challenges associated with traditional recombinant protein expression. The production of recombinant proteins involves gene design, protein expression, and purification. Compared with traditional protein extraction methods, recombinant protein technology offers advantages such as high production efficiency, superior purity, and reduced contamination risk (Baker et al. 2002; Jayakrishnan et al. 2024). Prokaryotic systems, such as *E. coli* , are widely used owing to their low cost and high yield. However, *E. coli* expression systems face challenges such as the formation of inclusion bodies and the lack of post‐translational modifications (Francis and Page 2010). The GAL1 tag is a low‐cost and safe option for improving recombinant protein purification while maintaining biological activity. In this study, GAL1 was used as a fusion tag to develop a novel purification system based on a chitin matrix, and the binding affinity and biological activity of the GAL1–MELT fusion protein were evaluated.

The GAL1 fusion tag demonstrated a strong binding affinity for chitin. Previous studies have shown that GAL1 can bind to chitosan membranes, enhance cell adhesion and growth, and disrupt the peritrophic membrane in diamondback moth larvae (Chen et al. 2009). These effects might be contributed to by the interaction between GAL1 and chitin mediated by CRD, which binds to N‐acetylglucosamine units. This binding ability supported the idea of using chitin as a purification material. Chitin has been utilized for affinity‐based purification owing to its non‐toxic, inert, and stable properties (Pina et al. 2014). In this study, a pQE_GAL1–Linker–MELT expression vector was constructed, and M15 *E. coli* was used to express the fusion protein. Purification results showed that the binding of GAL1–MELT to chitin was dose‐ and time‐dependent. In addition, SDS effectively eluted the bound proteins, likely by altering the electrostatic interactions between GAL1 and chitin. Although enzymatic cleavage of the linker sequence was not tested in this study, it may further improve the purity of the recovered melittin, suggesting the potential versatility and practicality of the GAL1 tag.

Bioactivity assays showed that the GAL1–MELT fusion protein retained its biological functions. Previous studies have suggested that large protein tags, such as LacZ, can interfere with the activity of target proteins owing to their dimeric properties when the tags remain uncleaved. Additionally, potential drawbacks associated with enzymatic tag removal have been reported. Specifically, the use of proteases to recover target proteins may result in unintended cleavage of the target protein, leading to a loss of functional activity or the generation of unexpected impurities, which could adversely impact subsequent purification and analysis processes (Kimple et al. 2013). Therefore, we conducted bioactivity assays without removing the GAL1 tag.

GAL1–MELT exhibited strong inhibitory effects against both Gram‐negative ( *E. coli* ) and Gram‐positive ( *B. subtilis* ) bacteria, with minimum inhibitory concentrations (MICs) of 6.25 μg/mL against *E. coli* and 25 μg/mL against *B. subtilis* . These concentrations were significantly lower than those of the fusion proteins with other tags. For instance, Zhou et al. (2020) reported that melittin with a glutathione‐S‐transferase (GST) tag exhibited MICs ranging from 41.0–43.5 μg/mL against *E. coli* (Zhou et al. 2020). A study of a melittin‐SUMO fusion protein showed MICs of 16 μg/mL against *E. coli* and 32 μg/mL against *B. subtilis* (Chen et al. 2021). These findings support the conclusion that GAL1–MELT exhibits superior antibacterial activity compared to other tags. GAL1 itself also exhibited a weak antibacterial effect on *E. coli* . However, studies on the antibacterial effects of related galectin proteins remain limited. Only GAL4 and GAL8 have been reported to induce the death of a specific *E. coli* strain (Stowell et al. 2010). This highlights the potential of the galectin protein family to function as antibiotics, but their mechanism of action remains unexplored.

**FIGURE 7:**
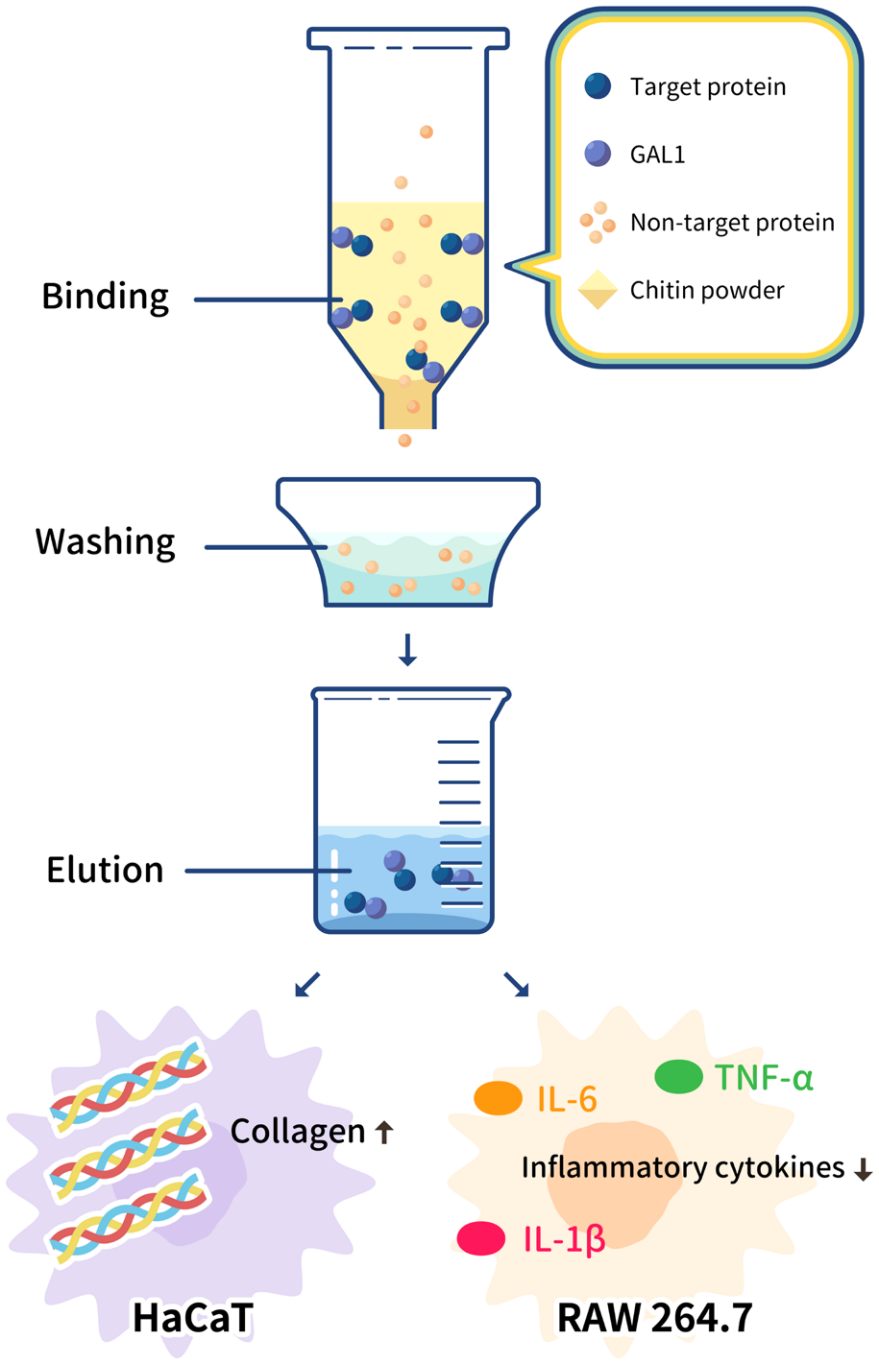
Chitin column purification process and bioactivity assays. A diagram showing the chitin column purification process and an overview of the bioactivity assays.

GAL1–MELT significantly reduced the expression of inflammatory cytokines *TNF‐α*, *IL‐1β*, and *IL‐6* in RAW 264.7 macrophages, with notable suppression observed after only 4 h (Figure 4). In previous studies where the protein tag was not removed, GST fusion proteins retained their anti‐inflammatory activity even without tag cleavage. A similar finding was observed in an anti‐inflammatory protein from the flatworm, *Schistosoma japonicum*. Additionally, the presence of the GST tag was found to decrease the negative effects of MELT‐induced cell cytotoxicity (Rayahin et al. 2014; Hu et al. 2009). Without removal of its large‐size protein label, the GAL1–MELT fusion protein displayed similar anti‐inflammatory effects to those observed with MELT only, indicating that the function of MELT was not interrupted by the GAL1 tag. Furthermore, GAL1 has the advantages of smaller size and lower cost compared to GST (Karav et al. 2017). Remarkably, GAL1 itself also displayed weak but significant anti‐inflammatory effects. This aligns with findings from mammalian studies, including research on murine paw edema and ocular inflammation (Zanon Cde et al. 2015; Rabinovich et al. 2000). In collagen production assays, the GAL1 tag exhibited no influence on MELT function (Figure 5B). GAL1–MELT increased type I collagen expression in HaCaT keratinocytes without significantly inducing allergenic gene expression, demonstrating its safety and potential for therapeutic applications. However, limitations might still exist. GAL1 is a multi‐function protein produced in mammals. The binding affinity of GAL1 to other substances on the cell surface might be a potential drawback (Stillman et al. 2006). Therefore, although removal of the GAL1 tag is not necessary for purified protein activity, further applications should still consider the side effects of the GAL1 tag itself. The modified GAL1 or the use of a specific chitin recognition domain (CRD) alone might be suitable alternatives. The schematic summary of GAL1‐MELT and chitin‐based purification system and bioactivity assays in this study was shown in Figure 7.

MELT is a 26‐amino‐acid antimicrobial peptide (AMP). Since GAL1–MELT retains its bioactivity, GAL1 fusion and chitin‐based purification could be applied to other AMPs. With this application, GAL1‐AMP could potentially facilitate AMP selection or target microbe selection. A recent review suggested that artificial intelligence could aid AMP selection (Brizuela et al. 2025); however, the current number of available peptides remains insufficient for deep‐learning models. Our findings offer new insights into bridging this data gap for future research.

The GAL1 fusion protein, produced cost‐effectively using our chitin‐based method, holds potential for vaccine production. This allows for efficient acquisition of the protein of interest, which can then be injected into host animals for antibody generation. Building on a previous study that designed a recombinant viral protein, the receptor‐binding domain of Omicron BA.1 spike protein (RBD‐Omicron) (Kalyoncu et al. 2025), combining our GAL1 tag and chitin‐based method could further reduce costs in future industrial applications.

The results of this study confirmed the advantages of the GAL1 fusion tag system in terms of purification efficiency and cost‐effectiveness (Table 2). The purification efficiency obtained using a chitin‐based matrix was comparable to or higher than that obtained using conventional Ni‐NTA affinity columns. Furthermore, the cost of chitin (0.2 $/g) was substantially lower than that of Ni‐NTA agarose (12.09 $/mL), supporting the economic feasibility of this system, particularly for large‐scale applications (Casadidio et al. 2019). Mild elution conditions using SDS reduced the risk of structural damage to the protein, representing an improvement over the harsher conditions required for Ni‐NTA‐based purification systems. Sodium dodecyl sulfate is also capable of denaturing protein. To mitigate this effect, we remove SDS by commercial kit before all protein activity assays. This approach successfully retained protein activity after elution. This approach could also be included for mass production on an industrial level. To summarise for scalability, the GAL1 fusion tag and chitin‐based purification system offer an economically viable and efficient approach for large‐scale protein production. However, in the present study, the achieved purity was only around 27% (Figure 3A). While this performance surpassed that of the Ni‐NTA column, further optimization—particularly of elution methods—is necessary to achieve higher purity levels.

**TABLE 1:** Primer sequences.

Primer	Sequence
*GAL1*	(F) 5′‐actggatccatggcctgtggtctggtcgcaagca‐3′ (R) 5′‐actctgaattcctcaaaggccacacacttgatcttg‐3′
*MELT*	(F) 5′‐actatgaattcttagttcctcgtggtagtatgaaattcttagtcaacgttgc‐3′ (R) 5′‐atcccgggttagtggtgatggtgatgatgaccctgttgcctcttacgtttaatc‐3′
*DsRed*	(F) 5′‐actacgaattcttagttcctcgtggtagtatggcctcctccgagaacgtc‐3′ (R) 5′‐actaagcttctacaggaacaggtggtggcggc‐3′
*TNF‐⍺*	(F) 5′‐gccaatggcatggatctcaaag‐3′ (R) 5′‐cagagcaatgactccaaagt‐3′
*IL‐1β*	(F) 5′‐tgtgaaatgccaccttttga‐3′ (R) 5′‐gtagctgccacagcttctcc‐3′
*IL‐6*	(F) 5′‐gaaatcgtggaaatgag‐3′ (R) 5′‐taggtttgccgagtaga‐3′
*RAW‐18S*	(F) 5′‐agcgaccaaaggaaccataa‐3′ (R) 5′‐ctcctcctcctcctctctcg‐3′
*Fos*	(F) 5′‐ggggcaaggtggaacagtta‐3′ (R) 5′‐agttggtctgtctccgcttg‐3′
*FosL1*	(F) 5′‐gccttgtgaacagatcagcc‐3′ (R) 5′‐agtttgtcagtctccgcctg‐3′
*HaCaT‐18S*	(F) 5′‐cacgcaagaagatccatcgc‐3′ (R) 5′‐ccggagcttgtgattcctgg‐3′

**TABLE 2:** Comparison of affinity tag technologies. Since some of the affinity resins listed are reusable, actual costs may be less.

Comparison of affinity tag technologies					
Tag	Size (aa)	Resin	Eluting agent	Cost ($/10 mg, 20 μL)	Source or reference
GAL1	155	Chitin	SDS	0.02	Tokyo Chemical Industry (TCL)
HIS	6	Ni‐NTA	Imidazole	21	Lichty et al. 2005
HA	9	HA	HA‐peptides	320	Sigma‐Aldrich
GST	218	GST	Glutathione	36	Lichty et al. 2005
FLAG	8	FLAG	FLAG‐peptides	1045	Lichty et al. 2005
V5	14	V5	V5‐tag protein	262	Sigma‐Aldrich

Despite these promising results, the potential limitations of this study should be considered. The binding efficiency of proteins to chitin matrices may be affected by physicochemical properties such as protein size, charge, and structural conformation (Mitchell and Lorsch 2015; Tang et al. 2024). Proteins with different characteristics may exhibit suboptimal binding or elution, necessitating modifications to purification conditions. For example, additional binding domains could be introduced for proteins with lower binding affinity, or the composition of the elution buffer could be adjusted to improve efficiency and flexibility (Tang et al. 2024). Future research should systematically investigate these factors to enhance the stability and adaptability of the chitin‐based purification system and further validate its application across different types of recombinant proteins. For example, this system could be tested for the purification of high molecular‐weight or multifunctional proteins, with assessments of their structural integrity and biological activity. Additionally, the implementation of enzymatic cleavage steps to obtain tag‐free purified proteins could further enhance the quality and applicability of biopharmaceuticals (Elsner et al. 2025). In conclusion, this study presents a cost‐effective and efficient approach for recombinant protein purification, providing new opportunities for biotechnological and industrial applications, supporting the scalable production of recombinant proteins, and expanding their potential utility in medical and skincare products.

## Author Contributions

**Yao‐Kuang Tseng:** methodology, software, data curation, investigation, formal analysis. **Yun‐Heng Lu:** software, data curation, writing – review and editing, formal analysis. **Yun Liu:** software, investigation, data curation. **Zhi‐Wei Weng:** formal analysis, data curation. **Yu‐Tzu Lin:** investigation, methodology. **Chih‐Hsuan Tsai:** methodology. **Yueh‐Lung Wu:** data curation, software, formal analysis, investigation, funding acquisition, writing – review and editing, writing – original draft, project administration. **Rong‐Nan Huang:** software, project administration, formal analysis, supervision, data curation, investigation, funding acquisition, writing – review and editing.

## Disclosure

Code Availability: Software applications are mentioned in the methods, and programs are publicly available.

## Conflicts of Interest

The authors declare no conflicts of interest.

## Supporting information


Data S1.

## Data Availability

The datasets generated during and/or analysed during the current study are available from the corresponding author on reasonable request.
