# Supplementary material for: A Novel Chitin‐Based Purification System Using GAL1 Fusion Tags: Enhancing Recombinant Protein Production While Retaining Biological Activity
Source: Microb Biotechnol. 2025 May 15;18(5):e70157. doi: 10.1111/1751-7915.70157 (PMC12079018; doi:10.1111/1751-7915.70157)
Supplement: Supplementary file 1 — Data S1. [file MBT2-18-e70157-s001.docx]

**A Novel Chitin-Based Purification System Using GAL1 Fusion Tags: Enhancing Recombinant Protein Production While Retaining Biological Activity**

Yao-Kuang Tseng^1^, Yun-Heng Lu^1^, Yun Liu, Zhi-Wei Weng^1^, Yu-Tzu Lin^1^, Chih-Hsuan Tsai^2^, Yueh-Lung Wu^1,3＊^and Rong-Nan Huang^1,3＊^

**Supplementary Figures**


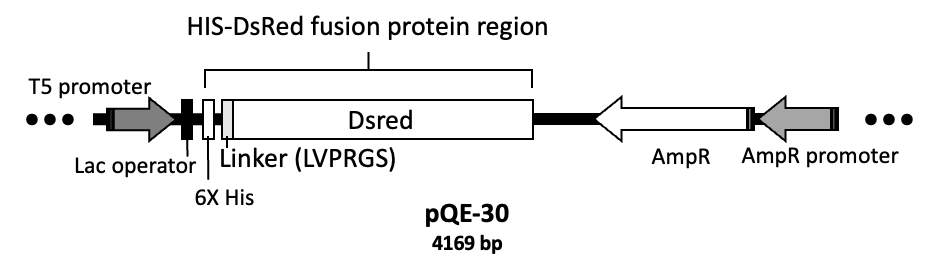


**Supplementary figure 1.** Follow diagram showing the genetic structure of the pQE_HIS–DsRed construct.

**Supplementary figure 2. Assessment of protein activity after chitin-based purification of GAL-dsRed fusion protein.** Data was presented in folds of fluorescence intensity relative to PBS group, which was set as background. BSA was set as negative control. His-DsRed was set as positive control. The dilution number from 1 to 5 indicated highest to lowest concentration of each protein.
